# Supplementary material for: Bacterial autolysins trim cell surface peptidoglycan to prevent detection by the Drosophila innate immune system
Source: eLife. 2014 Apr 1;3:e02277. doi: 10.7554/eLife.02277 (PMC3971415; doi:10.7554/eLife.02277)
Supplement: Supplementary file 1. — DOI: http://dx.doi.org/10.7554/eLife.02277.016 [file elife-02277-supp1.docx]

**Supplementary File 1: Strains and plasmids used in this study**

| **Strains** | **Relevant characteristics** | **Origin** |
| --- | --- | --- |
| ***S. aureus*** |  |  |
| RN4220 | Restriction deficient derivative of *S. aureus* NCTC8325-4 strain capable of receiving foreign DNA | R. Novick |
| NCTC8325-4 | MSSA *S. aureus* strain | R. Novick |
| MW2 | CA-MRSA *S. aureus* strain | (Baba et al., 2008) |
| NCTCΔ*oatA* | NCTC8325-4 *oatA* null mutant; *oat* encodes a *O*-acetyltransferase that determines PGN resistance to  lysozyme (Bera et al., 2005) | This study |
| NCTCΔ*fmtA* | NCTC8325-4 *fmtA* null mutant; *fmtA* is involved in the modulation of the major autolysin and in the synthesis of PGN (Qamar and Golemi-Kotra, 2012) | This study |
| NCTCΔ*arlR* | NCTC8325-4 *arlR* null mutant; *arlR* encodes a regulator of the expression of autolysins and other proteins (Liang et al., 2005) | This study |
| NCTCΔ*atl* | NCTC8325-4 *atl* null mutant; *atl* encodes a major staphylococcal autolysin (Oshida et al., 1995) | This study |
| NCTCΔ*tagO* | NCTC8325-4 *tagO* null mutant ; *tagO* encodes to a glycosyltransferase required for the first step of WTA biosynthesis | (Atilano et al., 2010) |
| NCTCΔ*tarS* | NCTC8325-4 *tarS* null mutant *tarS* is required for the attachment of β-*O*-GlcNAc residues to WTA (Brown et al., 2012) | This study |
| NCTCΔ*dltA* | NCTC8325-4 *dltA* null mutant; *dltA* is required for the production of teichoic acids with attached D-alanyl esters (Peschel et al., 1999) | This study |
| NCTCΔ*atl*Δ*tagO* | NCTC8325-4 lacking *atl* and *tagO* genes | This study |
| MW2∆*atl* | MW2 *atl* null mutant | This study |
| AM^H265A^ | NCTC8325-4 encoding Atl protein with mutation H265A in the amidase domain. | This study |
| GL^E1128A^ | NCTC8325-4 encoding Atl protein with mutation E1128A in the glucosaminidase domain. | This study |
| Atl^H265A/E1128A^ | NCTC8325-4 encoding Atl protein with mutations H265A and E1128A | This study |
| ***S. pneumoniae*** |  |  |
| R36A | Non-encapsulated laboratory strain | (Avery et al., 1944) |
| R36AΔ*lytA* | R36A *lytA* null mutant; *lytA* encodes a major pneumococcal autolysin (Mosser and Tomasz, 1970) | This study |
| R36AΔ*lytA*p*lytA* | R36A *lytA* null mutant complemented with replicative plasmid to express LytA | This study |
| ***L. lactis***  LL108 | RepA^+^ MG1363, Cm^r^ | (Leenhouts et al., 1996) |
| ***E. coli*** |  |  |
| EC101 | *E. coli* cloning strain with  *repA* from pWVO1 integrated in the chromosome | (Law et al., 1995) |
| DH5α | *recA endA1 gyrA96 thi-1 hsdR17 supE44 relA1 ϕ80 ΔlacZ*ΔM15 | Lab stock |
| DC10B | Δ*dcm* in the DH10B background; Dam methylation only | (Monk et al., 2012) |
| BL21(DE3) | *F–ompT gal dcm lon hsdSB(rB-mB-) λ(DE3 [lacI lacUV5-T7 gene 1 ind1 sam7 nin5])* | Lab stock |
| **Plasmids** |  |  |
| pMAD | *E. coli* – *S. aureus* shuttle vector with a thermosensitive origin of replication for Gram-positive bacteria; Amp^r^; Ery^r^; LacZ^+^ | (Arnaud et al., 2004) |
| pORI280 | Integrative vector which replicates in strains containing RepA; Ery^r^; LacZ^+^ | (Leenhouts et al., 1996) |
| pΔ*oatA* | pORI280 with *oatA* up and downstream regions | This study |
| pΔ*fmtA* | pORI280 with *fmtA* up and downstream regions | This study |
| pΔ*arlR* | pMAD with *arlR* up and downstream regions | This study |
| pΔ*atl* | pMAD with *atl* up and downstream regions | This study |
| pΔ*tarS* | pMAD with *tarS* up and downstream regions | This study |
| pΔ*dltA* | pMAD with *dltA* up and downstream regions | (Atilano et al., 2011) |
| pΔ*tagO* | pMAD with *tagO* up and downstream regions | (Atilano et al., 2010) |
| pAM^H265A^ | pMAD encoding a truncated Atl protein with mutation H265Ain AM domain | This study |
| pGL^E1128A^ | pMAD encoding a truncated Atl protein with mutation E1128Ain GL domain | This study |
| pBCSLF001 | High-copy-number vector, containing the −10 constitutive promoter of sigA from S. pneumoniae, Tet^r^ | (Henriques et al., 2011) |
| p∆*lytA* | pORI280 with *lytA* up and downstream regions | This study |
| p*lytA* | pBCSLF01 expressing *S. pneumoniae* LytA | This study |
| pGL100 | *E. coli* replicative plasmid expressing *S. pneumoniae* LytA. Amp^r^ | (Garcia et al., 1987) |
| pET28a(+) | *E. coli* expression vector. Km^r^. | Novagen |
| pET-AMR_1_R_2_R_3_GL | pET28a(+) expressing His6-AMR_1_R_2_R_3_GL protein. | This study |
| pET-AMR_1_R_2_ | pET28a(+) expressing His6-AMR_1_R_2_ protein. | This study |
| pET-R_3_GL | pET28a(+) expressing His6-R_3_GL protein. | This study |
| pET-AM^H265A^ | pET28a(+) expressing His6-AM(H265A)R_1_R_2_ protein. | This study |
| pET-GL^E1228A^ | pET28a(+) expressing His6-R_3_GL(E1228A) protein. | This study |
|  |  |  |

Avery OT, Macleod CM, McCarty M. 1944. Studies on the chemical nature of the substance inducing transformation of pneumococcal types: induction of transformation by a desoxyribonucleic acid fraction isolated from pneumococcus type III. *The Journal of Experimental Medicine* **79**:137–158.

Baba TF, Takeuchi M, Kuroda H, Yuzawa K, Aoki A, Oguchi Y, Nagai N, Iwama K, Asano T, Naimi H, Kuroda L, Cui K, Yamamoto, Hiramatsu K. 2002. Genome and virulence determinants of high virulence community acquired MRSA. *Lancet* **359**:1819–1827.

Bera A, Herbert S, Jakob A, Vollmer W, Gotz F. 2005. Why are pathogenic staphylococci so lysozyme resistant? The peptidoglycan O-acetyltransferase OatA is the major determinant for lysozyme resistance of Staphylococcus aureus. *Molecular Microbiology* **55**:778–787.

Brown S, Xia G, Luhachack LG, Campbell J, Meredith TC, Chen C, Winstel V, Gekeler C, Irazoqui JE, Peschel A, Walker S. 2012. Methicillin resistance in Staphylococcus aureus requires glycosylated wall teichoic acids. *Proceedings of the National Academy of Sciences of the United States of America* **109**:18909–18914.

Law J, Buist G, Haandrikman A, Kok J, Venema G, Leenhouts K. 1995. A system to generate chromosomal mutations in Lactococcus lactis which allows fast analysis of targeted genes. *Journal of Bacteriology* **177**:7011–7018.

Liang X, Zheng L, Landwehr C, Lunsford D, Holmes D, Ji Y. 2005. Global regulation of gene expression by ArlRS, a two-component signal transduction regulatory system of Staphylococcus aureus. *Journal of Bacteriology* **187**:5486–5492.

Monk IR, Shah IM, Xu M, Tan MW, Foster TJ. 2012. Transforming the untransformable: application of direct transformation to manipulate genetically Staphylococcus aureus and Staphylococcus epidermidis. mBio3:e00277.

Mosser JL, Tomasz A. 1970. Choline-containing teichoic acid as a structural component of pneumococcal cell wall and its role in sensitivity to lysis by an autolytic enzyme. *The Journal of Biological Chemistry* **245**:287–298.

Oshida T, Sugai M, Komatsuzawa H, Hong YM, Suginaka H, Tomasz A. 1995. A Staphylococcus aureus autolysin that has an N-acetylmuramoyl-L-alanine amidase domain and an endo-beta-Nacetylglucosaminidasedomain: cloning, sequence analysis, and characterization. *Proceedings of the National Academy of Sciences of the United States of America* **92**:285–289.

Peschel A, Otto M, Jack RW, Kalbacher H, Jung G, Gotz F. 1999. Inactivation of the dlt operon in Staphylococcus aureus confers sensitivity to defensins, protegrins, and other antimicrobial peptides. *The Journal of Biological Chemistry* **274**:8405–8410.

Qamar A, Golemi-Kotra D. 2012. Dual roles of FmtA in Staphylococcus aureus cell wall biosynthesis and autolysis. *Antimicrobial Agents and Chemotheraphy* **56**:3797–3805.
