# Supplementary material for: Bacterial autolysins trim cell surface peptidoglycan to prevent detection by the Drosophila innate immune system
Source: eLife. 2014 Apr 1;3:e02277. doi: 10.7554/eLife.02277 (PMC3971415; doi:10.7554/eLife.02277)
Supplement: Supplementary file 2. — DOI: http://dx.doi.org/10.7554/eLife.02277.017 [file elife-02277-supp2.docx]

**Supplementary File 2: Primers used in this study**

| **Primer** | **Sequence (5´-3´)*** |
| --- | --- |
| P1_oatA | GAGGATCCCAAAGCACAAGGTTTAGGTG |
| P2_oatA | TGCCATGGTCCATGTTAATAAACGCCC |
| P3_oatA | TGCCATGGACGATGGAAACACATGCTAC |
| P4_oatA | GCGAGATCTGATCAGTGAATAAACCGCTC |
| P5_fmtA | TATGGATCCGCTTTCATATTGACTCTCAC |
| P6_fmtA | GCCATATACATGTTATATCTTCTATATCATTTGATAATTGCCTCAC |
| P7_fmtA | GTGAGGCAATTATCAAATGATATAGAAGATATAACATGTATATGGC |
| P8_fmtA | TGGAGATCTTTGGGTTTTCATCTTTACTG |
| P9_arlR | CATGCCATGGCCTAAAGTGTCGTAAGGG |
| P10_arlR | GTCACGATCGTATGGTTTTAACTTTAAAATTTGCGTCATTTGTACACC |
| P11_arlR | GGTGTACAAATGACGCAAATTTTAAAGTTAAAACCATACGATCGTGAC |
| P12_arlR | TGGAGATCTGTTCGTAATTCATGTGACG |
| P13_atl | GGAAGATCTTCCGAAACAATGAAATCACTTAGC |
| P14_atl | GATCCTATGTTCATGTTGCTCTATTTATTACTCCTAAC |
| P15_atl | GTTAGGAGTAATAAATAGAGCAACATGAACATAGGATC |
| P16_atl | CGGAATTCCGCATATAAGGTACTATCAAACG |
| P17_tarS | GCGAGATCTCAGATCAAACAAGAGATGG |
| P18_tarS | CTCACAATGATTTGAGGGCATTTATATACCTCTCCCACTTTGACTAC |
| P19_tarS | GTAGTCAAAGTGGGAGAGGTATATAAATGCCCTCAAATCATTGTGAG |
| P20_tarS | CATGCCATGGTGGAAAATCGACATCGTCTTC |
| P25_dltA | GCAGATCTGAATGTATATATTTGCGCTGATG |
| P26_dltA | GTAAAATCACCATATGGAATCATATTAAGTCTCCCTCATTAGAACTC |
| P27_dltA | GAGTTCTAATGAGGGAGACTTAATATGATTCCATATGGTGATTTTAC |
| P28_dltA | GCGAATTCCGAAACGTTTGTAACGATCG |
| P29_AtlAM | CGGAGGATCCGCGAAAAAATTCAATTACAAACTACC |
| P30_AtlAM | TAGCTGTATCAGCAACTACGATACCTTCAGGACGACCTACGCCG |
| P31_AtlAM | GTATCGTAGTTGCTGATACAGCTAATGATCGTTCGACGATAAATG |
| P32_AtlAM | GCCTGAATTCGAGCAACACCGTTTAATGATGAAACTG |
| P33_AtlGL | GCGAGGATCCGTTTATTTATACGGTACTATTAATAACCGC |
| P34_AtlGL | CCGTTACCTGTCGCTAATAGGGCATGTGAGATAAGATAAACTTC |
| P35_AtlGL | CATGCCCTATTAGCGACAGGTAACGGTACTTCTCAATTAGCGAAAG |
| P36_AtlGl | CGCTGAATTCGTCAATGTTTATCGGAAGTCGAATTAGAC |
| P41_LytA | GCCGGATCCTTTAATTTCTTCTGCATCAAGG |
| P42_LytA | GTTGTTTTAATTGATAAGGAGTAGAATATGTAATAATGGAATGTCTTT  CAAATC |
| P43_LytA | CATATTCTACTCCTTATCAATTAAAACAAC |
| P44_LytA | GGTAAGAATTCGCCACAGTTTAAACATTTC |
| P45_LytA | GGCGCTAGCATGGAAATTAATGTGAGTAAATTAAG |
| P45_LytA | GCGAGATCTTTATTTTACTGTAATCAAGCCATC |
| Pexp1 | CCAGGATCCGCTTCAGCACAACCAAGATCAG |
| Pexp2 | CGTGGATCCGCTTATACTGTTACTAAACC |
| Pexp4 | CCAGTCGACTTATTTATATTGTGGGATGTCG |
| Pexp_stop1 | GCAGTAGCACAACCAAAAACAGCTGTAtaaGCTTATACTGTTACTAAAC  CACAAACG |
| Pexp_stop2 | CGTTTGTGGTTTAGTAACAGTATAAGCttaTACAGCTGTTTTTGGTTGTGC  TACTGC |
| Pexp_  H265A1 | GGTCGTCCTGAAGGTATCG TAGTTgcaGATACAGCTAATGATCGTTCGACG |
| Pexp_  H265A2 | CGTCGAACGATCATTAGCTGTATCtgcAACTACGATACCTTCAGGACGACC |
| Pexp_  E1228A1 | CTTATCTCACATGCCCTATTAgcaACAGGTAACGGTACTTCTC AATTAGC |
| Pexp_  E1228A2 | GCTAATTGAGAAGTACCGTTACCT GTtgcTAATAGGGCATGTGAGATAAG |
| Drs(+) | GTACTTGTTCGCCCTCTTCG |
| Drs(-) | TTAGCATCCTTCGCACCAG |
| Tbp (+) | GGCAAAGAGTGAGGACGACT |
| Tbp (-) | GAGCCGACCATGTTTTGAAT |
|  |  |

* Restriction sites are underlined
